# Assessment of psychotropic medications prescribing pattern in Gebretsadik Shawo General Hospital, South West Ethiopia

**DOI:** 10.11604/pamj.2023.45.165.30374

**Published:** 2023-08-17

**Authors:** Jafer Siraj

**Affiliations:** 1 Department of Pharmacy, School of Applied Natural Science, Adama Science and Technology University, Adama, Ethiopia

**Keywords:** Psychotropic, medications, prescription, pattern, Ethiopia

## Abstract

**Introduction:**

due to the widespread prescription of antipsychotic medications, their usage is cumulative. Evidence on the trends of medication use in Ethiopia and other parts of the world is lacking. The scant information on prescription trends and medication usage suggests that drug use is generally not sensible in both industrialized and emerging nations. So, the aim of this study was to assess the psychotropic medications prescribing pattern in Gebretsadik Shawo General Hospital, South West Ethiopia.

**Methods:**

from June 1^st^ to July 31^st^, 2019, a cross-sectional study on prescriptions for psychiatric drugs was conducted at Gebretsadik Shawo General Hospital. Using systematic random sampling, prescription records were obtained from the pharmacy dispensing book. Version 21 of the statistical program for social science was used to code and analyze the data.

**Results:**

the study included 355 prescription records containing psychotropic drugs in total. The bulk of those taking the psychotropic medication were aged 20 to 49. The most often administered classes of drugs remained antipsychotic, followed by tricyclic antidepressants, antiepileptics, anxiolytics/sedatives, anticholinergic and selective serotonin reuptake inhibitors. The most often ordered antipsychotic medication, which included 102 (23.18%) medications, was chlorpromazine. Tricyclic antidepressants, which included 56 medicines (12.73%) and 24 medications (5.45%), included amitriptyline and imipramine.

**Conclusion:**

the results of this investigation showed that psychiatrists preferred traditional psychotropic medications, such as Antipsychotic tricyclic, antidepressants (TCAs) and phenothiazines, in high amounts possibly because these medications were readily available in this hospital and their prices suited patients' needs. Health care workers' interdisciplinary relationships and coherence would improve for the benefit of patients and services of higher quality.

## Introduction

A significant contributor to irrational medication usage is irrational prescribing. Poor prescribing practices result in dangerous and ineffective treatments, illness aggravation or prolonging, patient discomfort and harm, and increased expenditures [1,2]. Medications are essential for maintaining and restoring health. The use of generic names of essential drugs is critical for the management of the syndrome, according to World Health Organization (WHO) [3]. Investigating discrepancies in medication use from evidence-based recommendations has required analyzing antipsychotic drug prescribing practices. The concurrent use of multiple antipsychotics in schizophrenic patients has been repeatedly cited as an issue. Although this may be reasonable for some individuals, the prescribing of such medications is still debatable [4-6]. The reported rates range from 5% to over 90% [7-9]. These vast variations may be brought about by evolving prescribing practices over time, regional variations related to cultural and traditional prescribing practices across nations, patient groups, and treatment settings [10-12]. For instance, patients treated at ambulatory clinics are classified as having relatively low levels of polypharmacy [13-18]. Any drug that has the potential to affect the mind, emotions, or behavior" is referred to as a psychotropic drug. Antidepressants, anxiolytic/hypnotics (mainly benzodiazepines to treat anxiety and insomnia), and antipsychotics are the three primary groups of psychotropic medications used. Anticonvulsants and stimulants are two other psychotropic groups. The main effects of psychotropic medications, which are frequently used to treat mental problems, are on the brain. These medications not only help patients' ailments, but they are also known to have long-term, potentially dangerous side effects on the central nervous system if not utilized properly [19]. Because of the numerous complex factors and inconsistent prescribing patterns, prescribing psychiatric medications differs from prescribing other medications [20]. According to research, bipolar African Americans were more likely than demographically similar white patients to receive first-generation antipsychotics (SGAs) for therapeutic management despite the risk of neuroleptic-induced tardive dyskinesia [21]. Prescription patterns are mostly useful for highlighting potential high-risk components that need additional careful review [22]. One of the primary components of the health care system, medicines plays a crucial role in resolving lives when utilized appropriately. The risk of adverse effects would increase with the amount of medication each prescription. This is frequently done more carelessly in developing countries because it is harder to detect and document adverse drug effects [23].

For practically all psychiatric disorders, the current treatment guiding concept of mentally competent persons recommends using monotherapy first. The difference between the recommendations supported by data and thus the actual practice in the care of schizophrenia disorders was correctly described by Stahl as “the dirty little secret” of psychiatry [24]. This dispute becomes primarily pure in the management of individuals with schizophrenia disorders when combination therapy is prohibited in clinical practice. Due to the mixing of antipsychotics with psychiatric medications from other medication categories, the course of treatment may include many antipsychotic drug combinations [25]. In this regard, a surge in the use of combination practice has been noted [26,27]. Unquestionably, a development in polypharmacy has been noted among illnesses that are affected by treatment resistance [28]. Polypharmacy is more common as the disease progresses and becomes more severe [29]. Due to the widespread use of antipsychotic medications, its usage is cumulative [30]. Evidence on the trends of medication use in Ethiopia and other parts of the world is lacking. The scant information on prescription trends and drug use suggests that drug use is generally not sensible in both industrialized and emerging nations [31]. To ensure that medications are administered correctly in terms of dosage and time, prescribing actions are essential. It will make sense to understand the circumstances around prescription error if you are familiar with Ethiopia. The promotion of knowledge about the selection of medications according to the quality treatment standards and from the Essential Medicines List frequently improves the prescribing practices among psychiatrists. The reduction of concurrent sedative-hypnotic prescriptions is a specialty area for prescriber training. Also, the prescribers would be strengthened to monitor the patients' compliance with the prescribed medications and to record them on the case pages. Such steps will promote pharmaceutical use that is reasonable and, over time, improve the standard of healthcare. This study was created to assess the patterns of psychotropic medication prescribing and, consequently, the prevalence of a problem with psychotropic medication prescribing in Gebretsadik Shawo General Hospital between June 1^st^ and July 31^st^, 2019.

## Methods

**Study area and period:** the study was conducted at Gebretsadik Shawo General Hospital, southwest Ethiopia regional state, from June 1^st^, 2019 to July 31^st^, 2019. Gebretsadik Shawo General Hospital is one of the general Hospitals in southern Ethiopia. It is found in Bonga town which is located 444 km Southwest of Addis Ababa, the capital city of Ethiopia. The hospital has various units including the inpatient ward with a total of 120 beds. It has 52 technical and 103 administrative staffs. The hospital serves the people of Bonga town, the administrative town of Kafa zone, and 10 woredas under its administration as a general hospital.

**Study design:** on prescription records containing psychotropic medications, a cross-sectional analysis was conducted. Throughout the data collecting period, prescriptions containing psychiatric drugs were collected from the psychiatry pharmacy and examined. The prescriptions' supporting documentation was altered to finish a modified data gathering instrument. The study population consisted of prescription papers containing psychotropic drugs that were systematically chosen from the source population. The source population consisted of a total one-year supply of prescription papers for psychotropic drugs in every pharmacy at Gebretsadik Shawo General Hospital. From January 2017 to January 2018, every prescription for psychiatric medications in the hospital's pharmacy database was included. Both prescriptions for substances other than psychiatric medications and those for psychotropic medications taken outside of the study period were disallowed.

**Sample size and sampling technique:** the sample size was decided by means of single population proportion formula [32]. Meanwhile, the source population was less than 10,000 (4644) so reduction method was used to compute the right sample size. So, 355 prescription papers were pinched to collect required evidence. Systematic random sampling method was used to choose/to select the necessary sample.

**Data collection tools and procedures:** data were collected using data collection checklist developed by reviewing similar studies with some modifications [33-35]. The questionnaire addressed the basic demographic characteristics, a pattern of prescribing error for psychiatric medications as well as a pattern of prescribing trends (often prescribed psychotropic medications, average dosage per prescription, and quantity of pills ordered by brand/generic name). To ensure the validity of the study of the instruments, the data collection checklist was pretested before actual data collection. Minor modifications were made based on the feedback obtained from the pilot testing. To help with the data collection, two trained pharmacy professionals were hired. Supervisors double-checked the accuracy and consistency of the data collected.

**Data collection:** to collect information from each prescription paper, a data collecting tool was created. The tool included information about the patient (including sex, age, and card number), the prescriber (including a signature and official stamp), and the drug prescribing pattern (including medication group, number of prescriptions, dose, frequency, method, amount/course of treatment, etc.). Throughout the data collection process, the author reviewed the consistency and completeness of the assembled data every day.

**Data analysis:** the collected data were coded, entered, and analyzed using a Statistical Package for Social Sciences (SPSS, version 21). Descriptive statistics such as frequency, percentage, and mean were used to summarize demographic characteristics, the most frequently prescribed psychotropic drugs irrespective of their categories, Prominence of suitability of necessities in prescriptions agreeing to the “Guide to good prescribing”, amount and proportion of unsuitable prescribing and /or omissions of necessities in prescriptions attended as per the WHO “guide to good prescribing”, psychotropic drugs name prescribed by generic, brand and abbreviation and number of psychotropic drugs prescribed per prescription.

**Ethics approval and consent to participate:** ethical clearance was obtained from School of Pharmacy, College of Medicine and Health Sciences, Mizan-Tepi University and was forwarded to the hospital officers (Ref. no DP0102/17). Furthermore, written informed consent to participate in the study was secured from the study participants before data collection. To ensure the confidentiality of participants, the name and address of the study participants was not recorded in the data collection format.

## Results

Investigations were conducted on 355 prescription documents containing psychotropic medications. Almost 22 (6.20%) and 21 (5.91%) of the total prescriptions lacked crucial information, such as the sex and age of the patients, respectively. Table 1 displays the age and gender distribution of the patients who got psychotropic medication. Those between the ages of 20 and 39 made up the largest age group, numbering 197 (55.49%). As per the this finding, adult population groups had generally greater rates of psychiatric problems than other population group. Among these age groups, 86 (25.75%) of the prescriptions were given to women, while 111 (33.23%) of the prescriptions were given to men (Table 1). Small number of psychotropic medications was prescribed for geriatrics population groups which accounted of 10 (2.99%). Majority of psychotropic prescriptions were subjected to males population which accounted of 213 (63.78%). This finding revealed that there is high prevalence of psychotic disorders in men than women. The patients received a total of 440 prescriptions for psychotropic medications. Antipsychotics made up the majority of patients' therapies, accounting for 231 (52.5%), followed by antidepressants at 91 (20.68%) and antiepileptics at 63 (14.31%) (Table 2). From antipsychotics drug category, chlorpromazine was the most frequently prescribed drug which accounted for 44.15% (n=102), followed by thioridazine and haloperidol which constituted for 62 (26.84%) and 59 (24.24%), respectively. Amitriptylline was the most frequently prescribed antidepressants which accounted for 56 (61.54%), followed by imipramine and fluoxetine which constituted for 24 (24.37%) and 11 (12.09%), respectively. The lowest percentages of prescription medicine classes were seen for anxiolytics (n=33) and anticholinergics (n=22) in the study area. Antipsychotic tricyclic, antidepressants (TCAs), antiepileptics, anxiolytics/ sedatives, anticholinergic, and selective serotonin reuptake inhibitors (SSRIs) were the classes of medication that were most often administered.

**Table 1:** age and sex distribution of studied subjects

Classification of Patients by Age	Male		Female		Total	
	No.	%	No.	%	No.	%
1-19	73	21.86	25	7.49	98	29.34
20-39	111	33.23	86	25.75	197	58.98
40-59	23	6.89	6	1.80	29	8.68
60	6	1.80	4	1.20	10	2.99
Total	213	63.78	121	36.24	334	100

**Table 2:** the most frequently prescribed psychotropic drugs irrespective to their categories

Drugs categories	Specific drugs	Frequency	Percent
**Antipsychotic**	Chlorpromazine	102	23.18
	Thioridazine	62	14.09
	Haloperidol	59	13.41
	Trifluoperazine	8	1.82
	Total	231	52.5
**Antidepressant**	Amitriptylline	56	12.73
	Imipramine	24	5.45
	Fluoxetine	11	2.50
	Total	91	20.68
**Antiepileptics**	Phenobarbitone	32	7.27
	Phenytoin	18	4.09
	Carbamazepine	7	1.59
	Sodium valporate	6	1.36
	Total	154	14.31
**Anxiolytics**	Diazepam	33	7.50
**Anticholinergics**	Trihexyphenidyl	22	5.00
**Total**		440	

**Figure 1:**
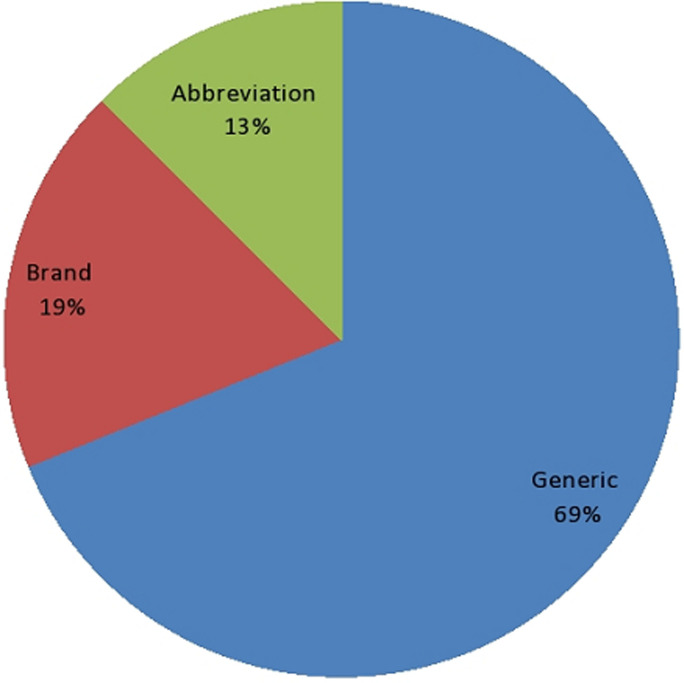
psychotropic drugs name prescribed by generic, brand and abbreviation

Chlorpromazine was the most often prescribed anti-psychotic, accounting for 102 prescriptions (23.18%), followed by thioridazine, accounting for 62 (14.09%), and haloperidol, accounting for 59 (13.41%). Trifluoperazine was administered at the smallest dose of the 8 (1.82%) antipsychotic drug classes. Tricyclic antidepressants were the most frequently prescribed antidepressants to patients, followed by fluoxetine 16 medications, according to antidepressant categories (Table 2). In the group of tricyclic antidepressants, which included 56 drugs (12.73%) and 24 drugs (5.45%), respectively, imitriptyline and imipramine were present. The two most commonly prescribed psychotropic medications from the antiepileptic drug categories were phenobarbitone (32 pharmaceuticals, or 7.27% of total), and phenytoin (18 drugs, or 4.09%) (Table 2). The fewest quantities of prescription anxiolytics and anticholinergic drugs were diazepam (33 tablets) and trihexyphenidyl (22 tablets), respectively. The prescriptions were evaluated in light of the World Health Organization's [36] prescription guidelines. It offers step-by-step instructions for the rational prescribing procedure. The medication name (brand, generic, or abbreviation), the date, the patient's and prescriber's identification, the presence of the prescriber's authorized stamp, the presence of the medication's route of administration, the presence of its pharmaceutical form, the dosage, the frequency of its administration, and the length of the course of treatment were evaluated as a result. Out of the 440 medications prescribed at the mental health treatment facility, 303 (60.86%) were ordered by generic name, 82 (18.64%) by brand name, and 55 (12.50%) by abbreviation (Figure 1). We placed orders for both brand-name and generic amitriptyline. Thirty-eight (38) of the total number of amitriptyline drugs were ordered under the name Amirol®. Trihexyphenidyl (Artane®), 33 prescriptions, and trifluoperazine (Modicate®), 11 pills, were the other psychotropics that the brand recommended. These drugs were categorized as anticholinergics and antipsychotics, respectively. 78 (17.73) of the 102 chlorpromazine pills were ordered using an abbreviation.

**Figure 2:**
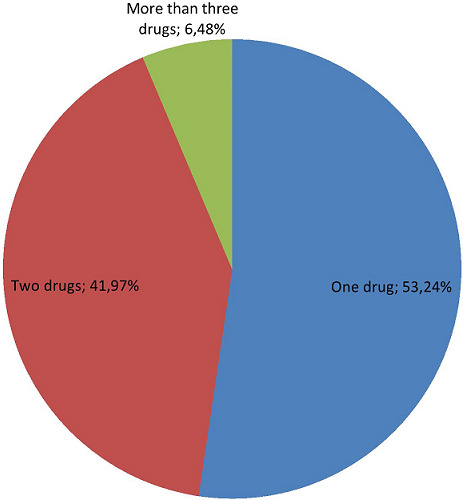
number of psychotropic drugs prescribed per prescription

Out of the total prescription papers, about 302 (68.64%) prescription papers were contained patient card number and the rest 53 (14.93%) prescription papers was not contained patient card number. Majority of prescription papers were contained patient sex which accounted for 333 (93.80%). About 21(5.91%) of prescription papers were lacked age of the patients which is important parameter to calculate the dose of medication especially in geriatrics and pediatrics patient. In a similar vein, a doctor's signature appears on about 292 (82.25%) prescription orders. On the other hand, just 146 (41.13%) of the prescription orders contained a licensed stamp, and as a result, 189 (53.24%) of the prescriptions did not accept this stamp. 260 prescriptions (73.24%) lacked a date of prescription, whereas 195 (54.93%) had a chosen date of medication (Table 3). Considering the number of medicines per prescription, 186 (48.44%) of the 355 prescriptions (or the bulk of them) covered just one drug (Figure 2). Also, 149 (41.97%) of the prescription papers contained two medications. 23 (6.48%) of the remaining prescription papers included three medications. There were 1.77 prescriptions for medications on average for each visit. According to the WHO prescribing criteria, a total of 248 errors and omissions, as well as 28 instances of inappropriate prescribing, were identified in the current investigation (Table 4). Drug dosage, dosage type, frequency of administration, site of administration, amount administered, and management strategy were all subject to omission errors. Moreover, 28 erroneous prescriptions were found, including ones with the wrong dosage, wrong dosage form, wrong frequency of administration, and incorrect dosage and/or course of treatment. Accordingly, out the total drugs prescribed, the correct dosages of the drugs were omitted in 100 (22.73%) of medications whereas the correct dosage forms of the drugs were omitted in 122 (27.73%). Correct frequency of administration and correct route of administration was omitted in 155 (35.22%) and 120 (27.27%), respectively. About 126 (28.64%) omission errors were observed in terms of correct quantity or course of treatment of the psychotropic drugs (Table 4). In general, the majority of omission errors were related with frequency of administration which accounted for 155 (35.22%), followed by quantity or course of treatment and dosage forms omission errors which accounted for 126 (28.64%) and 122 (27.73%), respectively.

**Table 3:** prominence of suitability of necessities in prescriptions agreeing to the “guide to good prescribing”

Prerequisite parameters		No of prescription	percent
Card no	Yes	302	68.64
	No	53	14.93
Patient sex	Yes	333	93.80
	No	22	6.20
Age of patient	Yes	334	94.08
	No	21	5.91
Prescriber signature	Yes	292	82.25
	No	63	17.75
Authorized stamp	Yes	146	41.13
	No	189	53.24
Date of prescription	Yes	195	54.93
	No	260	73.24

**Table 4:** amount and proportion of unsuitable prescribing and /or omissions of necessities in prescriptions attended as per the WHO “guide to good prescribing”

		Frequency	Percent
Correct dosage	Yes	332	75.45
	No	8	1.82
	Omitted	100	22.73
Correct dosage Form	Yes	313	71.14
	No	5	1.14
	Omitted	122	27.73
Correct frequency of administration	Yes	281	63.86
	No	4	0.91
	Omitted	155	35.22
Correct route of administration	Yes	318	72.27
	No	2	0.45
	Omitted	120	27.27
Correct quantity or course of treatment	Yes	305	69.31
	No	9	2.05
	Omitted	126	28.64

## Discussion

As the initial treatment option is significant for therapeutic effectiveness and patient compliance, the research of psychotropic drug prescription in mental patients is vital. Antipsychotic drugs were the most often prescribed psychotherapy drug in the current study, followed by antidepressant drugs. This report closely resembles an investigation done on the prescribing of psychotherapy drugs in Trinidadian mental health facilities, which revealed that patients of African origin took more antipsychotics while they were also more frequently diagnosed with schizophrenia and that the proportion of male patients was much higher for patients of African origin [37]. Also, nearly identical to a study conducted on long-term mental patients living in Rotterdam sheltered housing facilities, 25% of patients received a polypharmacy of psychotherapy medications, and the majority of patients (79%, n=255) took antipsychotic medications [38]. According to a recent study [39], women are more likely than males to take psychiatric drugs. A female doctor is substantially more likely to prescribe psychiatric medications of any kind to women than a male doctor [39]. On the other hand, the specific study did not take into account the evidence about the patient diagnosis or the severity of the symptoms. In this study, men received an average of more psychiatric medication prescriptions than women. This contradicts a research conducted in Finland that revealed no gender differences [40]. Also, in contrast to the current study, women were administered psychiatric medicines at a teaching hospital in Western Nepal more often than men [41]. From early adolescence through the middle of their 50s, women have a lifetime rate of depression that is 1.7-2.7 times higher than that of men [42]. Even though despair can strike at any age, adults between the ages of 18 and 29 experience the highest rates of major depression within any given year [43]. According to a study, primary sadness has been reported to occur 20.4% more frequently in women than in men between the ages of 65 and 80 [44]. Men are more likely than women to develop schizophrenia, but men often experience the illness at a younger age. Men typically experience their first episode in their early 20s, but for females it typically happens in their late 20s to early 30s [45,46]. This study found that chlorpromazine was the phenothiazines medicine most frequently administered, in contrast to a study conducted in Trinidad that found sulpiride to be the most commonly used antipsychotic [37]. This discrepancy results from the medicine sulpiride not being available in the study hospital.

Tricyclic antidepressants were the class of antidepressants that were prescribed the most frequently in the current study, which is consistent to other studies conducted elsewhere [47,48]. Patients may prefer TCAs over SSRIs because of the former's inconsistent and unreliable accessibility, the latter's higher cost, and the fact that psychiatrists feel more at ease giving conventional drugs [37]. In this study, SSRI prescriptions were much lower than TCA prescriptions, and the findings are consistent with those from Trinidad [37] and Western Nepal [41]. The advantage of SSRIs appears to be their reduced frequency of side effects (absence of sedative, anticholinergic, and hypotensive effects); their wider therapeutic index, which reduces the risk of overdosing; and their once-daily dosing, which may increase patient compliance [49-51]. In the current study, 303 medications (68.86%) and 82 (18.64%) were ordered by brand name and generic name, respectively. This pattern is the subject of an analysis conducted at a specialist hospital affiliated with Jimma University, where generic prescriptions made up 87.1% of all prescriptions and brand-name prescriptions made up 10.3% [52]. Moreover, our findings are less significant than those of a study conducted in Pondicherry, where 88.54% of drugs were prescribed using their generic names [53]. This study's observation of brand prescribing may be the result of a lack of knowledge or carelessness towards generic prescribing. According to a study, ordering pharmaceuticals by generic names saves money and increases the likelihood that patients would take their meds [54]. For all drugs, it is strongly advised to utilize the generic name [36]. Consequently, using generic names, particularly in underdeveloped nations like Ethiopia, may help to lower the price of medications. In this survey, it was found that more prescriptions-186, or 48.44 percent had one medication in them. In contrast to the results published from Western Nepal (13.72%) [39] and Italy (40%) [47], only 23 patients (6.48%) reported taking three or more drugs. In the current study, 1.77 prescriptions for medications were made on average each visit. This finding is consistent with the WHO recommendations for prescription, which state that an average prescription contains two medications [36]. Yet when we compare our findings to those of other studies, we find that they fall far short of those from studies conducted in Italy and the Netherlands, which reported averages of 2.7 and 4.6 medications per encounter, respectively [47,48]. The complete proof needed for a given drug may be absent as the number of medications per prescription increases due to a lack of locations, which could result in medication mistake. The biggest percentage of prescriptions 189 (53.24%) in this survey were found to be written without a prescriber's authorization stamp. The main reason for this is that not being able to identify the prescriber could lead to drug abuse and unauthorized use, both of which could cause serious harm to community health organizations [52]. World health Organization applauds the requirement that a prescription include the patient's ID and the date of the prescription [36] in order to prevent patients from accessing drugs without a prescription. In this analysis, an average of one omission per prescription was found. This may be the result of the prescribers' carelessness or neglect throughout the prescription process. This issue might imply that formulas can be changed at the point of dispensing. This could have an impact on desired and anticipated pharmacokinetic properties, such as absorption, which depends on the medication's ability to dissolve [52,55]. In order to prevent the development of antimicrobial resistance and achieve the desired optimal healing outcome, the WHO supports making this information mandatory in a prescription. Prescribers would therefore be familiar with the pharmaceutical dosage form and quantity of the commonly used medications. They can stay focused on the actual medications as a result.

**Limitation:** due to the lack of a specific prescription paper for psychiatric drugs, it might be challenging to distinguish between prescription papers containing psychotropic drugs and other prescriptions. This takes time and necessitates evaluating all given medications to separate psychotropic medications.

## Conclusion

The results of this investigation showed that psychiatrists preferred traditional psychotropic medications, such as TCAs and phenothiazines, in high amounts (possibly because these medications were readily available in this hospital and their prices suited patients' needs); they used anxiolytic and anticholinergic medications in moderate amounts. Since the primary treatment option is critical for therapeutic success and patient compliance, prescribing psychotropic medicines to mental patients should be done with caution

### What is known about this topic

- *Psychotropic medications prescribing pattern in different age groups has been reported especially in the developed countries and the developing countries including Africa;*
- *The prescribing trends of psychotropic medications and the incidence of psychotropic medicine prescribing problem have also been reported both in the developed and the developing countries*.

### What this study adds

- *Valuable results on the prescribing trends of psychotropic medications and prescribing errors for the scientific communities;*
- *Psychiatrists preferred traditional psychotropic medications, such as TCAs and phenothiazines, in high amounts;*
- *The WHO prescription guidelines have frequently been abused*.
